# Synergistic Chemopreventive Effects of a Novel Combined Plant Extract Comprising Gallic Acid and Hesperidin on Colorectal Cancer

**DOI:** 10.3390/cimb45060312

**Published:** 2023-06-05

**Authors:** Szu-Jung Chen, Jui-Hua Lu, Chih-Cheng Lin, Shao-Wei Zeng, Jia-Feng Chang, Yuan-Chiang Chung, Hsiang Chang, Chih-Ping Hsu

**Affiliations:** 1 Department of Radiation Oncology, Taoyuan General Hospital, Ministry of Health and Welfare, Taoyuan City 330, Taiwan; csj995@yahoo.com.tw; 2 School of Dentistry, College of Oral Medicine, Taipei Medical University, Taipei City 110, Taiwan; d225101001@tmu.edu.tw; 3 Department of Biotechnology and Pharmaceutical Technology, Yuanpei University of Medical Technology, Hsinchu City 300, Taiwan; lcc@mail.ypu.edu.tw (C.-C.L.); hchang@mail.ypu.edu.tw (H.C.); 4 Department of Medical Laboratory Science and Biotechnology, Yuanpei University of Medical Technology, Hsinchu City 300, Taiwan; allonc0331@gmail.com; 5 Division of Nephrology, Department of Internal Medicine, Taoyuan Branch of Taipei Veterans General Hospital, Taoyuan City 330, Taiwan; 6 Department of Nursing, Yuanpei University of Medical Technology, Hsinchu City 300, Taiwan; 7 Department of Surgery, Kuang Tien General Hospital, Taichung City 437, Taiwan; chung11753@ktgh.com.tw

**Keywords:** gallic acid, hesperidin, colorectal cancer, cell cycle, cancer stemness, spheroids

## Abstract

Background/Aim: Colorectal cancer (CRC) is the third most common cancer with a high mortality rate worldwide. Although gallic acid and hesperidin exert anticancer activity, synergistic effects of gallic acid and hesperidin against CRC remain elusive. This study aims to investigate the therapeutic mechanism of a novel combination of gallic acid and hesperidin against CRC cell growth, including cell viability, cell-cycle-associated proteins, spheroid formation, and stemness. Methods: Gallic acid and hesperidin derived from Hakka pomelo tea (HPT) were detected by colorimetric methods and high-performance liquid chromatography using ethyl acetate as an extraction medium. CRC cell lines (HT-29 and HCT-116) treated with the combined extract were investigated in our study for cell viability (trypan blue or soft agar colony formation assay), cell cycle (propidium iodide staining), cell-cycle-associated proteins (immunoblotting), and stem cell markers (immunohistochemistry staining). Results: Compared with other extraction methods, HPT extraction using an ethyl acetate medium exerts the most potent effect on inhibiting HT-29 cell growth in a dose-dependent manner. Furthermore, the treatment with combined extract had a higher inhibitory effect on CRC cell viability than gallic acid or hesperidin alone. The underlying mechanism was involved in G1-phase arrest and Cip1/p21 upregulation that could attenuate HCT-116 cell proliferation (Ki-67), stemness (CD-133), and spheroid growth in a 3D formation assay mimicking in vivo tumorigenesis. Conclusion: Gallic acid and hesperidin exert synergistic effects on cell growth, spheroids, and stemness of CRC and may serve as a potential chemopreventive agent. Further testing for the safety and effectiveness of the combined extract in large-scale randomized trials is required.

## 1. Introduction

Colorectal cancer (CRC) is the third most common cancer with a high mortality rate worldwide, and its prevention and treatment have emerged as issues of great importance [1]. The traditional Hakka pomelo tea (HPT) contains flavonoid polyphenols derived from Citrus species that exert pleiotropic effects on diverse disorders, e.g., CRC, diabetes, obesity, and inflammation due to microbial infection [2].

Furthermore, recent studies revealed that citrus-derived major phenolic acid (gallic acid) and flavonoids (hesperidin) exhibited a versatile role in anti-oxidation, anti-inflammation, anti-apoptosis, and anticancer effects [2,3]. The radical scavenging ability of hesperidin and its derivatives results from their structures which abound in hydroxyl groups [3]. Gallic acid has been reported to enhance anti-oxidant enzyme activities, regulating inflammatory cytokines and cytosolic calcium ion concentrations and inhibiting reactive oxygen species production [3]. In light of their antioxidant properties, HPT extracts comprising gallic acid and hesperidin may be developed as chemopreventive agents for CRC. Nonetheless, the optimal HPT extraction method to provide a CRC-preventive effect remains unknown. Herein, we attempted to optimize the extraction method to obtain the highest concentration of bioactive components (gallic acid and hesperidin) from HPT to treat CRC. Meanwhile, we not only examined their anti-CRC activities but also explored the potential therapeutic mechanisms, including cell viability, cell-cycle-associated proteins, spheroid growth, and stemness. Through investigating the CRC cell cycle and cancer cell stemness, we aim to develop a combination of gallic acid and hesperidin as a novel chemopreventive agent for CRC.

## 2. Materials and Methods

### 2.1. Materials

Roswell Park Memorial Institute (RPMI) medium 1640, fetal bovine serum (FBS), L-glutamine, trypsin, and antibiotics were purchased from Gibco Ltd. (Paisley, UK). Human CRC cell lines (HT-29 and HCT-116) were obtained from the Bioresource Collection and Research Center, Taiwan, and cultured in 90% RPMI medium 1640 with 10% heat-inactivated FBS. Folin–Ciocalteu reagent and standards such as gallic acid (98%), caffeic acid, myricetin, quercetin, catechin (98%), (-)-epicatechin (EC) (98%), (-)-epigallocatechin (EGC) (98%), (-)-epicatechin gallate (ECG) (98%), (-)-gallocatechin gallate (GCG) (98%), (-)-epigallocatechin gallate (EGCG) (95%), delphinidin, cyanidin, malvidin, peonidin, hesperidin, proteinase inhibitor cocktail, sodium orthovanadate, sodium fluoride, sodium pyrophosphate, Triton X-100, ammonia persulfate, N,N,N′,N′-tetramethyl ethylenediamine (TEMED), and Tween 20 were obtained from Sigma (St. Louis, MO, USA). Bicinchoninic acid (BCA) protein assay reagent was purchased from Pierce (Rockford, IL, USA). Acrylamide was obtained from Bio-Rad (Hercules, CA, USA). Polyvinylidene fluoride (PVDF) membrane (Immobilon-P) was purchased from Millipore (Bedford, MA, USA). Rabbit polyclonal antibodies to cyclin D1, cyclin E, Cip1/p21, Kip1/p27, β-actin, and goat anti-rabbit secondary antibody conjugated with horseradish peroxidase (HRP) were obtained from R&D Systems (Minneapolis, MN, USA). Anti-Ki-67 and -CD133 antibodies, secondary antibodies conjugated with multiple HRP and chromogen for immunohistochemical staining were from GeneTex (Hsinchu, Taiwan). Ten-year-stored HPT was purchased from the Peasant Association of Xinpu (Hsinchu, Taiwan).

### 2.2. HPT Extraction

Four different methods were employed for the extraction of HPT: conventional boiling water (HPT-BW), hot water reflux (HPT-HW), ethanol (HPT-Alc), and acetone/acetate/ethyl acetate (HPT-EA) extraction, mainly modified from our previous reports [4,5,6]. Briefly, crushed HPT blocks were mixed at a ratio of 1:10 *w*/*v* with different solvents such as distilled water, 70% ethanol, or 100 mM acetate buffer, pH 4.8, in water/acetone (30:70 *v*/*v*). The boiling water method was carried out by cooking the mixture of HPT and water sufficiently to reduce the volume of water to 25% of the original volume. The hot water reflux method was carried out by refluxing the mixture in a 70 °C water bath for 12 h. The other two methods involved reflux of the mixture as described above for 12 h at room temperature. All of the extracted mixtures were then separately filtrated through NO1 filter paper, and the filtrates were centrifuged at 3000 rpm for 30 min. The supernatants of HPT-BW and -HW were frozen at −80 °C. The supernatants of HPT-Alc and HPT-EA were concentrated until ethanol and acetone were not retained using a rotary evaporator under reduced pressure and a water bath at a temperature of <35 °C. The concentrated HPT-EA solution was extracted four times with an equal volume of ethyl acetate. The ethyl acetate layers were collected and concentrated using a rotary evaporator until ethyl acetate was not retained. The obtained solutions of HPT-Alc and HPT-EA were then frozen at −80 °C. Finally, all of the frozen solutions were lyophilized to become powders [6].

### 2.3. Measurement of Phytochemicals

The levels of total phenolics, flavonoids, and condensed tannin in the different HPT extracts obtained from the different extraction methods mentioned above were determined by colorimetric assay as reported previously [7]. The total content of phenolic compounds in the HPT extracts was then determined by a standard curve prepared with gallic acid and expressed in terms of milligrams of gallic acid equivalents per gram of solid extracts. Both flavonoids and condensed tannin were expressed as milligrams of catechin equivalents per gram of dry weight.

### 2.4. HPLC Analysis of Gallic Acid and Hesperidin

The gradient separating condition of HPLC was applied for HPT. Gallic acid and hesperidin were analyzed by HPLC using a Shimadzu SCL-LC 10A HPLC apparatus fitted with a SIL 10AD autosampler as reported previously [8]. Chromatography was performed with an ODS HYPERSIL (Thermo Scientific, Waltham, MA, USA) reverse-phase column (25 cm × 0.46 cm i.d., 5 μm) and a UV-VIS detector (Shimazu systems Co., Foster City, CA, USA). The mobile phase contained 0.4% phosphoric acid (solvent A) and acetonitrile (solvent B), with a linear gradient from A/B (80:20) to A/B (73:27) over 40 min with a flow rate of 1 mL/min. The detector was set at 280 nm.

### 2.5. Cell Proliferation Assay

CRC cells were plated at 100,000 cells per 60 mm tissue culture dish. After 18 h of culture, cells were treated with DMSO-dissolved HPT-BW, HPT-HW, HPT-Alc, and HPT-EA extracts (0, 25, 50, 100, or 200 μg/mL). At 24 h and 48 h, cells were collected by trypsinization and stained with trypan blue, and the numbers of cells in the suspensions were counted in duplicate using a hemocytometer. Data are the averages of three independent experiments. To examine the combined effects of gallic acid and hesperidin (the major components of the HPT extracts), CRC cells were plated in six-well tissue culture dishes as described above and treated with DMSO-dissolved gallic acid and hesperidin alone or in combination. After 48 h of treatment, the number of cells in each treatment group was counted using the method described above. The combination effect was calculated according to the equation described by Kern et al. [9]. According to the prior research, an expected value for cell survival, S-exp, was defined as the product of the survival observed for gallic acid alone and the survival observed for hesperidin alone: S-exp = (S-gallic acid) × (S-hesperidin) [9].

The actual survival observed under combination treatment with gallic acid and hesperidin was defined as S-obs. A synergistic ratio, R, was calculated as R = (S-exp)/(S-obs). Synergy was defined as any value of R greater than unity. An R value higher than unity indicated an additive effect, and an R value lower than unity revealed an absence of synergy.

### 2.6. Cell-Cycle Analysis

As described in a previous report [5], treated cells were collected by trypsinization and then fixed in 70% ethanol at −20 °C for at least 30 min. Fixed cells were reconstituted in phosphate-buffered saline (PBS) and then stained with 20 μg/mL propidium iodide and 10 μg/mL RNase A at 37 °C in the dark for 30 min. The cell cycle of the treated cells was examined by flow cytometry (Becton Dickinson, Franklin Lakes, NJ, USA), using FL-2A to score the DNA content of cells. The numbers of cells in the G1, S, and G2/M cell-cycle phases were determined using Modfit software and expressed as the percentage of total cells (Verity Software House, Inc., Topsham, ME, USA).

### 2.7. Immunoblotting

Treated cells were washed with ice-cold PBS and lysed in homogenization buffer (10 mM Tris-HCl at pH 7.4, 2 mM EDTA, 1 mM EGTA, 50 mM NaCl, 1% Triton X-100, 50 mM NaF, 20 mM sodium pyrophosphate, 1 mM sodium orthovanadate, and 1:100 proteinase inhibitor cocktail) on ice for 30 min. After centrifugation for 30 min at 100,000× *g* at 4 °C to remove insoluble materials, the protein concentration of the lysate was determined using a BCA protein assay kit. Proteins were then separated by sodium dodecyl sulfate polyacrylamide gel electrophoresis (SDS-PAGE); the resolved bands were electro-transferred to PVDF membranes using a semi-dry blot apparatus (Bio-Rad), and immunoblotting was performed by incubating PVDF membranes with 5% non-fat milk in Tris-buffered saline supplemented with Tween 20 (TBST, 10 mM Tris, pH 7.4, 150 mM NaCl, 0.2% Tween 20) for 1 h at room temperature to block residual free protein binding sites. The membrane was then incubated with different primary antibodies in 3% non-fat milk in TBST at 4 °C for 18 h. After repeated washing with TBST, the membrane was incubated with secondary antibodies conjugated with HRP. Immunoblots were developed using enhanced chemiluminescence, and the luminescence was visualized using a chemiluminescence detection system (Bio-Rad).

### 2.8. Spheroid Formation in Soft Agar

CRC cells were seeded in soft agar for in vitro tumorigenic growth essentially as described by Hsu et al. (2010) [7]. One thousand CRC cells were suspended in 2 mL molten 0.3% agarose, which was dissolved in condition medium containing 20% FBS. The suspension was quickly overlaid on a feeder agar layer which was made of 0.6% agarose in condition medium supplemented with 20% FBS in a 6-well plate. One milliliter complete medium with gallic acid or hesperidin or their combination was added onto the solidified agar, and the plates were incubated at 37 °C. The number of formed spheroids with a diameter of more than 0.5 mm was counted under a microscope at the indicated time. At the end of the experiment, the spheroids in soft agar were collected and fixed in 3% formalin for further tissue processing.

### 2.9. Tissue Processing and Immunohistochemical Staining

The formalin-fixed spheroids of CRC cells in soft agar were applied to tissue processing, embedded in molten paraffin wax, resected into 3 μm thin slices, and stained with antibodies for immunohistochemistry. The detailed methods were essentially described in a previous report [10]. The slices from different treated colonies were followed by incubation with anti-Ki-67 (cell proliferation indicator) and anti-CD133 (CRC stem cell marker) at the recommended dilutions, and the images were obtained using a digital pathology scanner. The strong positive cells under Ki-67 and CD133 staining were counted and expressed as the percentage among the total number of malignant cells in a colony. The data were averages from at least 5 different colonies.

### 2.10. Statistical Analysis

All data are the averages of three independent experiments and expressed as mean ± standard deviation (SD) unless stated otherwise. Differences between groups were calculated using the ANOVA test. A *p* value < 0.05 was considered statistically significant. All statistical analyses were performed using SPSS version 17.0 (SPSS, Inc., Chicago, IL, USA).

## 3. Results

### 3.1. EA Extract of HPT Contained the Most Abundant Polyphenols

As shown in Table 1, the dry weight of total phenols of HPT-BW, -HW, -Alc, and -EA extracts was 62.65 ± 4.13, 58.92 ± 2.88, 197.85 ± 13.41, and 325.16 ± 23.73 mg/g, respectively. The polyphenol content of the HPT-EA extract was approximately 6-fold that of both water extracts and 1.5-fold that of the HPT-Alc extract, suggesting EA extraction is the optimal method. However, there was no significant difference in total flavonoids between the HPT-EA extraction method and other extraction methods. Although traditional extraction of HPT in daily consumption is via the boiling water method, the phenolics are relatively lower in the HPT-BW extract than in the HPT-Alc and -EA extracts, implying that some useful compounds such as polyphenols may be lost in the traditional usage of HPT. The gallic acid and hesperidin contents in HPT-Alc and -EA were analyzed using a colorimetric assay, which showed greater contents of these two components in HPT-EA than in HPT-Alc (Table 2). Furthermore, we analyzed the phenolic species by using HPLC chromatograms. The results indicate that gallic acid and hesperidin are the two major bioactive components of HPT. The profiles of HPLC measurement are provided in Supplemental Figures S1 and S2.

### 3.2. HPT-Alc and HPT-EA Treatment Inhibited HT-29 Cell Growth in a Dose-Dependent Manner

**Figure 1:**
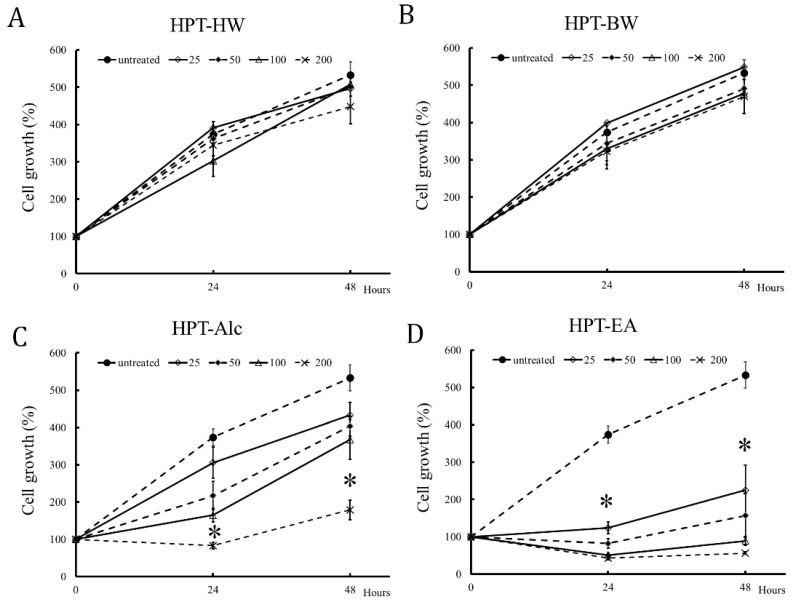
Anti-CRC activity of different HPT extracts in inhibiting HT-29 cell growth. HT-29 cells were plated at 100,000 cells per well in 6-well tissue culture plates and treated with different concentrations of extracts as indicated: (**A**) HPT-HW (hot water reflux), (**B**) HPT-BW (boiling water), (**C**) HPT-Alc (alcohol), and (**D**) HPT-EA (ethyl acetate). At 24 and 48 h, cells were trypsinized and stained with trypan blue, and the cell number was counted in duplicate using a hemocytometer. Data are the averages of three independent experiments and expressed as mean ± SD. The statistical results are shown as significant differences (* *p* < 0.05) from the control group. Abbreviations: CRC, colorectal cancer; HPT, Hakka pomelo tea; BW, boiling water; HW, hot water reflux; Alc, ethanol; EA, ethyl acetate.

As shown in Figure 1, the number of untreated HT-29 cells increased by approximately 5-fold after 48 h as compared with the baseline cells. HPT-BW or HPT-HW treatment showed only a slight influence on HT-29 cell growth, even when the treatment concentration was up to 200 μg/mL (Figure 1A,B). In contrast to the above two extracts, HPT-Alc and HPT-EA treatment inhibited HT-29 cell growth through gradually increasing concentrations (Figure 1C,D). Furthermore, HPT-EA exhibited an inhibition effect on HT-29 cell growth at a concentration of 25 μg/mL after 24 h of treatment (Figure 1D). Overall, the number of HPT-EA-treated cells was significantly lower than the baseline cell number at concentrations higher than 50 μg/mL after 24 h of treatment (Figure 1C,D). Notably, HPT-EA almost totally suppressed cell growth at concentrations of 100 and 200 μg/mL after 24 h treatment (Figure 1D). These positive results were correlated with the higher contents of bioactive polyphenols via the HPT-EA extraction method (Table 1 and Table 2), indicating that active components gallic acid and hesperidin may inhibit HT-29 growth.

### 3.3. HPT Retarded the G1 Phase of the Cell Cycle

**Figure 2:**
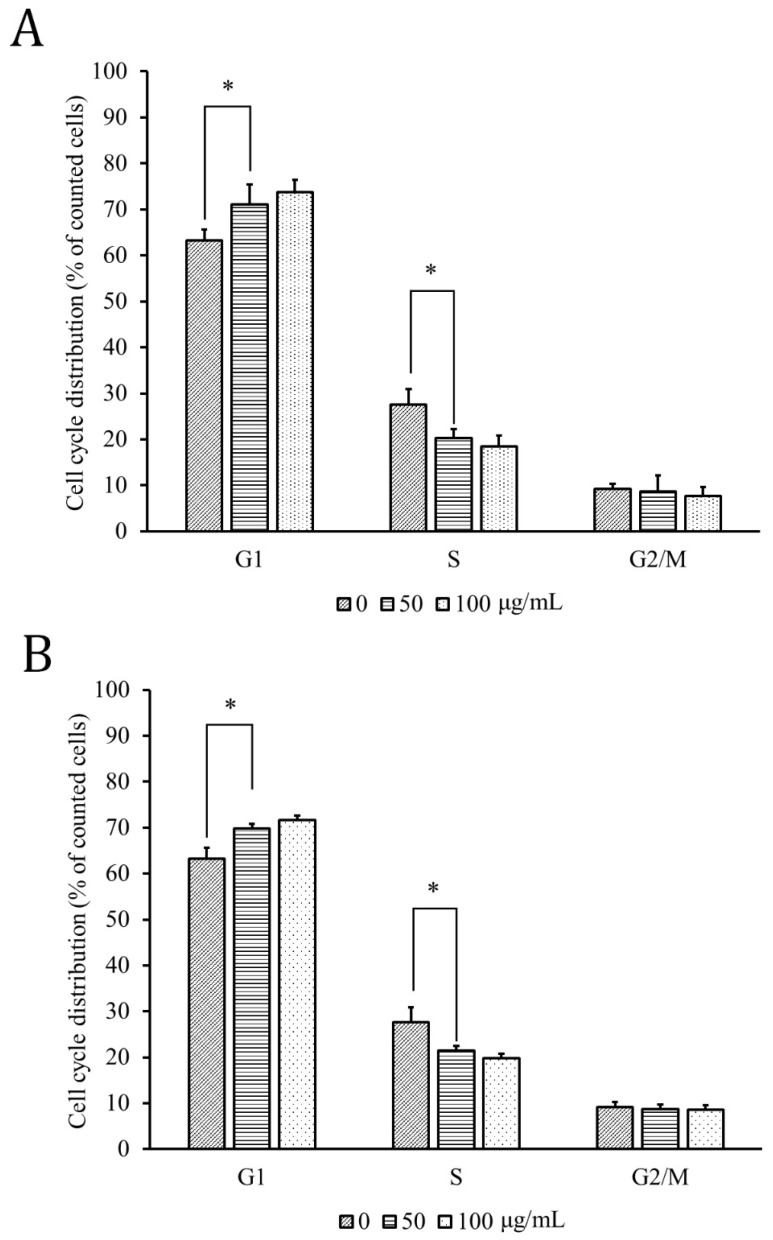
Cell-cycle arrest induced by treatment with HPT in HT-29 cells. HT-29 cells treated with different concentrations of HPT-Alc (**A**) or HPT-EA (**B**) as indicated were incubated at 37 °C for 48 h and then fixed in 70% alcohol and stained with propidium iodide, followed by flow cytometry analysis of the cell-cycle distribution of each treated group. Data are the average of three independent experiments and expressed as means ± SD. * *p* < 0.05.

The cell-cycle distributions of the HPT-Alc- and HPT-EA-treated HT-29 cells are shown in Figure 2. The percentage of G1-phase cells following 50 μg/mL and 100 μg/mL treatment with both extracts increased gradually from 62% of the untreated level to near 70%, while the S-phase cells correlatively decreased from 28% to lower than 19%. The percentage of G2/M-phase cells did not differ between untreated cells and HPT-treated cells, indicating that the influence of HPT treatment was on the G1 phase into the S phase transition.

### 3.4. HPT Treatment Attenuates Cyclins D1 and E and Accentuates Cip1/p21 Expression via G1-Phase-Controlling Protein Mechanism

**Figure 3:**
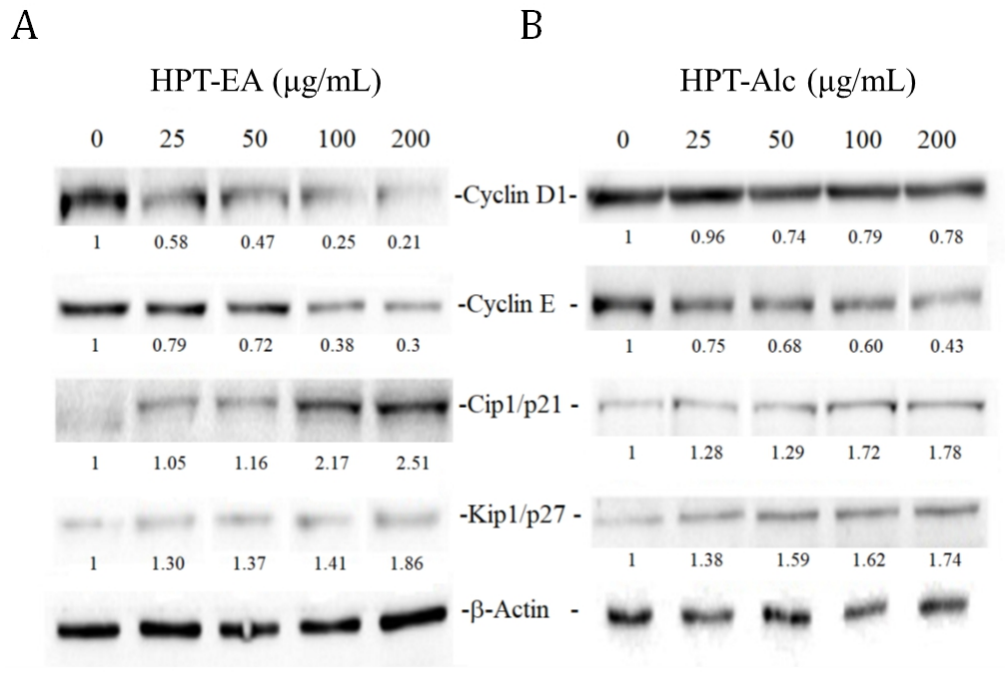
Expression levels of cell-cycle-associated proteins in HPT-treated cells. HT-29 cells were treated with different concentrations of HPT-EA (**A**) or HPT-Alc (**B**) as indicated and incubated at 37 °C for 48 h. The treated cells were lysed in lysis buffer, and the cell protein lysates were separated by SDS-PAGE, transferred to PVDF membranes, and immunoblotted to show proteins as indicated. The intensities of protein bands on the immunoblotting image were quantified using Image Lab software (Bio-Rad) according to the density of each band. The results were normalized to the intensity of β-actin as the reference band and are shown as the fold change of the untreated control. Abbreviations: HPT, Hakka pomelo tea; Alc, ethanol; EA, ethyl acetate.

We further verified the underlying mechanisms of G1-phase-controlling proteins of the cell cycle that were influenced by HPT-Alc and -EA in HT-29 cells. The levels of G1-phase-controlling proteins cyclins D1 and E, and cyclin-dependent protein kinase inhibitors Cip1/p21 and Kip1/p27, were determined by immunoblotting. As shown in Figure 3A, cyclins D1 and E in HPT-EA-treated cells gradually decreased to less than 0.3-fold the control level with increasing treatment concentrations of HPT. The level of Cip1/p21 was elevated under treatment at greater than 100 µg/mL HPT-EA to 2.17- to 2.51-fold that of the control, while the level of Kip1/p27 was elevated under treatment at greater than 25 µg/mL HPT-EA to 1.3- to 1.86-fold that of the control. HPT-Alc-treated cells also expressed gradually decreasing levels of cyclins D1 and E and increasing levels of Cip1/p21 and Kip1/p27; however, the level changes of these proteins were less obvious than those of HPT-EA-treated cells (Figure 3B).

### 3.5. Synergistic Inhibition Effect of Gallic Acid and Hesperidin on CRC Cell Proliferation

**Figure 4:**
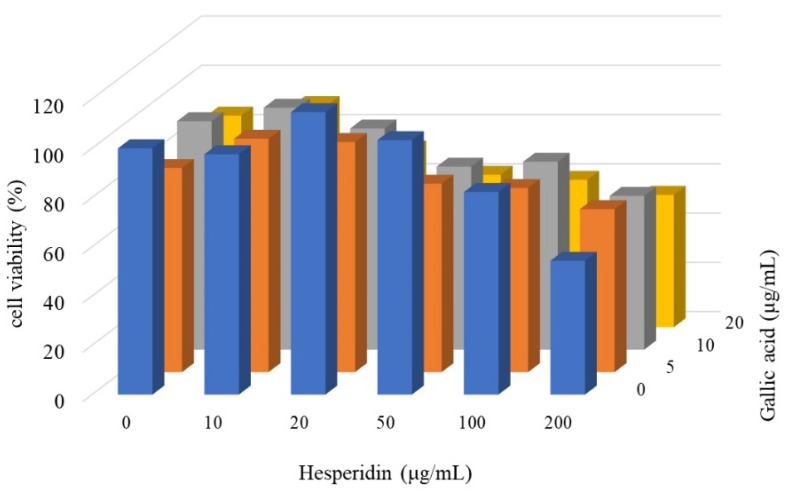
Effect of combined treatment with gallic acid and hesperidin on HT-29 cells. A total of 100,000 HT-29 cells per well in 6-well plates were treated with gallic acid and hesperidin, or both in combination, as indicated at 37 °C for 48 h. Viable cells were trypsinized, stained with trypan blue, and counted under a microscope. Cell viability was expressed as the percentage of that of untreated cells. Data are the average of three independent experiments.

HT-29 cells were treated with various concentrations of gallic acid and hesperidin alone or in combination, and the surviving cells were counted. As shown in Figure 4, the viability of the hesperidin-treated cells was almost not altered at lower than 100 μg/mL and was approximately 55% at 200 μg/mL. The viability of the gallic acid-treated cells was only slightly decreased to approximately 85% of that of untreated cells, even at 20 μg/mL (Figure 4). However, the viability of the HT-29 cells treated with a combination of these two compounds was decreased significantly to approximately 60% of that of untreated cells at 50 μg/mL hesperidin and 20 μg/mL gallic acid. Furthermore, the synergistic effects of combined treatment were apparently greater than unity, as the R values were 1.43, 1.37, and 1.29, respectively (Table 3).

### 3.6. Inhibition of In Vitro Tumor Growth by Combined Gallic Acid and Hesperidin Treatment

**Figure 5:**
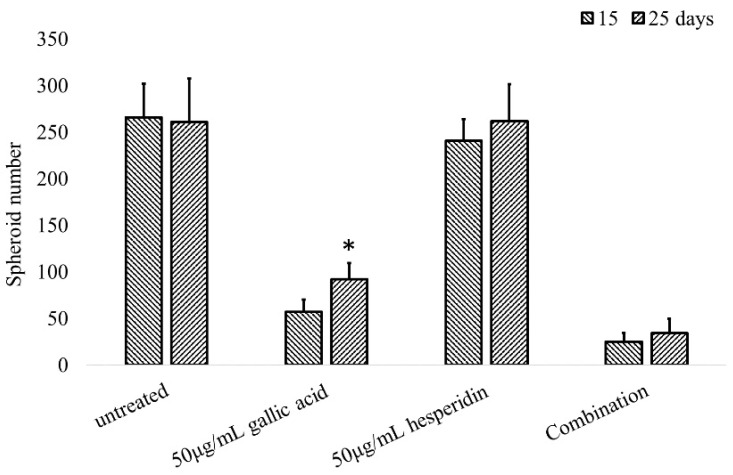
The combination effect of gallic acid and hesperidin on the in vitro tumorigenic growth of CRC cells. One thousand HCT-116 cells were suspended in soft agar as described in Materials and Methods and treated with different concentrations of gallic acid or hesperidin only, or their combination as indicated. The formed colonies with a diameter greater than 0.5 mm were counted under a microscope at the indicated time. Data are the averages of three independent experiments and expressed as means ± SD. The statistical results are shown as significant differences (* *p* < 0.05) between spheroid numbers at 15 and 25 days.

Although the combination effect of gallic acid and hesperidin, the active components of HPT, was obviously found in 2D-cultured HT-29 cells, the effect in the 3D tumors was not evaluated. Thus, we selected another CRC cell line, HCT-116, which is used for 3D spheroid formation assays for anti-CRC drug screening to mimic the in vivo tumorigenesis [11]. The low-density HCT-116 cells were first cultured in soft agar, which allowed the single HCT-116 cell to grow and became a tumor spheroid. The tumor-spheroid growth was inhibited by gallic acid treatment. The number of spheroids was only 57 after a 15-day treatment with gallic acid, whereas 266 and 241 spheroids were found in the untreated control and hesperidin-treated group on the 15th day (Figure 5). The combination treatment with gallic acid and hesperidin was effective in inhibiting the growth of spheroids, the number of which was only 25 on the 15th day of treatment. Intriguingly, the number of spheroids in the gallic acid treatment group was significantly elevated to 92 on the 25th day, whereas it reached 34 in the combination group. HCT-116 cells seem to be recurrent or resistant to gallic acid, and the combination with hesperidin could overcome this resistance.

### 3.7. Cell Division and Stemness in Gallic Acid and Hesperidin Combination-Treated Spheroids

**Figure 6:**
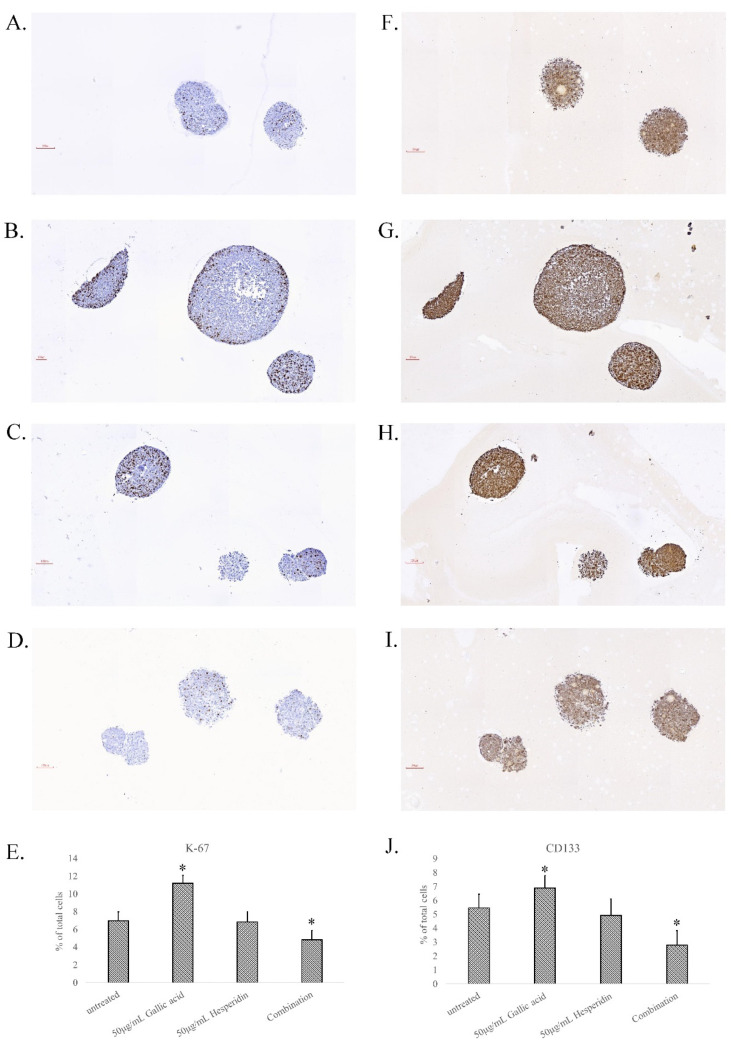
Cell division and stemness in the in vitro tumors of HCT-116 cells after treatment with gallic acid or hesperidin or their combination. The soft agar colonies as described in the Figure 5 legend were applied for tissue processing, and the resection slices of parafilm-embedded spheroids were stained with anti-Ki-67 antibody for indicating the division cells and anti-CD133 antibody for CRC stem cells by immunohistochemical staining. Representative images were captured of spheroids stained with anti-Ki-67 antibody from the untreated control (**A**), 50 μg/mL gallic acid (**B**), 50 μg/mL hesperidin (**C**), and the combination of gallic acid and hesperidin (**D**). The Ki-67-positive cells were counted and calculated as a percentage of the total tumor cells in the spheroid of the image (**E**). The images of CD133 stained spheroids from the untreated control (**F**), 50 μg/mL gallic acid (**G**), 50 μg/mL hesperidin (**H**), and the combination of gallic acid and hesperidin (**I**) are shown. The Ki-67-positive cells were counted and calculated as a percentage of the total tumor cells in the spheroid of the image (**J**). The statistical results are shown as significant differences (* *p* < 0.05) from the control group.

The proliferative potential of the HCT-116 spheroid cells in each treatment group was evaluated by immunohistochemical staining with an anti-Ki-67 antibody. Many nuclear dark/brown stains of spheroid cells were found in untreated (Figure 6A), 50 μg/mL gallic acid-treated (Figure 6B), and 50 μg/mL hesperidin-treated spheroids (Figure 6C), whereas only a few cells were strongly stained by the Ki-67 antibody in the combination group (Figure 6D). Ki-67-positive cells in each group were scored, and 6.98% and 6.84% of total tumor cells were calculated in the untreated group and hesperidin-treated group, whereas the proportions were 11.21% in the gallic acid-treated group and 4.84% in combination-treated spheroids (Figure 6E). Cancer stem cells are associated with drug resistance of CRC cells. The CRC stem cell marker CD133 was applied to each treated group of spheroids. The CD133 staining images of untreated (Figure 6F), 50 μg/mL gallic acid-treated (Figure 6G), and 50 μg/mL hesperidin-treated (Figure 6H), and combination-treated spheroids (Figure 6I) are shown. The strong positive CD133-stained cells were counted, and 5.46% and 4.94% of total tumor cells were calculated in the untreated group and hesperidin-treated group, whereas the proportions were 6.89% in the gallic acid-treated group and 2.79% in the combination-treated group (Figure 6J). The CRC stem cells seem to be abundant in gallic acid-treated spheroids and decreased after combination with hesperidin.

## 4. Discussion

In this study, our data showed HPT extract with an ethyl acetate medium exerts the most potent effect on inhibiting HT-29 cell growth in a dose-dependent manner. Moreover, we found that cell-cycle arrest was induced by treatment with HPT-Alc or HPT-EA in HT-29 cells. Intriguingly, HPT-Alc or HPT-EA treatment attenuated cyclins D1 and E and accentuated Cip1/p21 expression through the mechanism of G1-phase-controlling proteins. Notably, the combination treatment had a higher inhibitory effect on CRC cell viability than gallic acid or hesperidin alone, indicating a synergistic effect of these two compounds. The underlying mechanism was involved in G1-phase arrest and p21/Cip-1 upregulation that could attenuate HCT-116 cell proliferation (Ki-67), stemness (CD-133), and spheroid growth in a 3D formation assay mimicking in vivo tumorigenesis. To the best of our knowledge, this is the first study to reveal the synergistic suppression effect of gallic acid and hesperidin on CRC cell growth. Several novel findings deserve further discussion.

Our previous study revealed that alcohol or acetic acid/acetone followed by ethyl acetate extraction could be used to obtain rich polyphenol species from longan seeds [5]. In the present study, we extracted HPT using different extraction methods and evaluated the active components of each HPT extract. In different extraction methods, the contents of the two major components in HPT (gallic acid and hesperidin) were in parallel with their total polyphenols (Table 1 and Table 2). Indeed, the acetone/acetate method followed by the ethyl acetate extraction method could obtain the most abundant amount of gallic acid and hesperidin from HPT, serving as the optimal method (Supplemental Figures S1 and S2). Accordingly, we evaluated the anti-CRC activities of the above extracts, demonstrating HPT-EA exhibited the most potent activity in terms of suppressing CRC cell growth (Figure 1) and retarding the cell cycle in the G1-to-S phase transition (Figure 2). Our previous study also showed that the polyphenol content was closely correlated with the extracts’ anti-CRC activities in vitro [4,5,6,7]. As expected, our results for HPT polyphenols were in agreement with the previous results, implying that some polyphenol species in HPT may exert antiproliferation activity on cancer cells. Gallic acid has been demonstrated to inhibit cell proliferation in several cancer cells, including CRC [12,13,14,15]. Hesperidin appears to be a chemopreventive agent and is correlated with a lower risk of CRC [16,17,18,19]. One report revealed the controversial result that hesperidin could reduce the side effects and efficacy of cycloheximide for CRC in a mouse model [19]. However, another study showed that a hesperidin-rich plant decoction combined with 5-FU suppressed tumor growth in a mouse model [20]. In light of this, we combined gallic acid and hesperidin to treat CRC cells, and we observed a synergistic effect, indicating the anticancer activities of these two phenolic compounds and their active role in the anti-CRC activity of HPT.

Our previous reports revealed that polyphenol-rich extracts are directly involved in cancer cell growth inhibition by interfering with cell-cycle progression [5,6,7,8,21,22,23]. However, the influence on the cell-cycle phase of polyphenols from different sources was not consistent. Some extracts or phenolic species arrested cells in the S phase [5,7], while some did in the G2/M phase [6,22] and others did in the G1 phase [8,21]. The possible mechanisms appear to involve the expression of cyclins and their inhibitors. When the expression of cyclin A was suppressed, the S phase was arrested, whereas on suppression of cyclin D1 and/or elevation of Cip1/p21, the G1 phase was arrested. In the present study, the HPT polyphenols suppressed cyclin D1, increased the Cip1/p21 expression, and arrested CRC cells in the G1 phase, indicating that the HPT extract influences the G1-phase mechanisms to inhibit CRC cell growth (Figure 3 and Figure 4).

Although in vitro 2D-cultured cells showed an effective response to the combination treatment of gallic acid and hesperidin, similar phenomena did not seem to occur in 3D CRC spheroids. Originally, gallic acid could potentially inhibit HCT-116 cell growth into spheroids in soft agar, while hesperidin presented no effect on spheroid growth (Figure 5). The use of the combination of gallic acid and hesperidin to treat CRC cells limited spheroid growth more effectively than treatment with gallic acid only. When the culture duration was extended to 25 days, the number of spheroids in the gallic acid treatment group was significantly increased by approximately 2-fold compared to the number of spheroids at 15 days. There were more Ki-67-positive dividing cells in spheroids of the gallic acid group than in other groups. CD133, a stem cell marker of CRC, was also higher in the gallic acid group than in other groups (Figure 6). It is accepted that drug resistance of CRC cells is closely associated with the contents of cancer stem cells [24,25,26]. The CRC stem cells being abundant in gallic acid-treated groups may indicate the resistance of CRC cells to gallic acid treatment. Hesperidin has been reported to inhibit cancer stem cells, including CRC stem cells [26,27,28]. The use of the combination of hesperidin and gallic acid to treat CRC spheroids could inhibit spheroid growth more effectively than gallic acid only. This result was possible due to the inhibition of CRC stem cells by hesperidin, especially when combined with gallic acid.

Some limitations are recognized in our study. In our HPLC analysis of HPT, we can find that there is more than one peak in the flushing time interval of gallic acid and hesperidin. This suggests that HPT contains more than one compound in this interval, and the additional compounds may be the isoforms of gallic acid or hesperidin. The precision of quantification for these two components in HPT could be improved by liquid chromatography–mass spectrometry (LC-MS) in accordance with previous research [29,30]. In the subsequent experiments, it is necessary to analyze these compounds using the LC-MS method. Next, fundamental aspects such as the difference between cellular-level results and the complexity of absorption and distribution for medical treatment in an organism should be taken into account. Last but not least, it is important to be aware that further studies are required for this treatment to truly be preventive.

## 5. Conclusions

We employed optimal extraction methods to obtain the highest amount of polyphenols from HPT and demonstrated that their anti-CRC activity arose from their influence on the G1 phase of the cell cycle by enhancing the Cip-1/p21 expression and suppressing cyclin D1. The major bioactive ingredients in HPT were gallic acid and hesperidin, and the combination induced a synergistic inhibition effect on CRC cell proliferation (Ki-67) in 2D-cultured cells, 3D spheroid growth, and CRC stem cells (CD-133). In conclusion, the novel combination of gallic acid and hesperidin could serve as a promising chemopreventive agent or adjuvant treatment for CRC. Further testing for the safety and effectiveness of the combined extract in large-scale randomized control trials is required.

## 6. Patents

The Hakka pomelo tea (extracts of Lao-Yiou tea) has been approved for the application of inhibition of colorectal cancer as a patent in Taiwan Intellectual Property Office (patent number: I581800; publication date: 11 May 2017).

## Figures and Tables

**Table 1:** Comparison between different HPT extraction methods for phytochemicals.

	HPT-BW	HPT-HW	HPT-Alc	HPT-EA
Total phenols (mg/g)	62.65 ± 4.13	58.92 ± 2.88	197.85 ± 13.41 *	325.16 ± 23.73 *
Total flavonoids (mg/g)	67.42 ± 5.06	88.48 ± 9.41	89.46 ± 2.68	84.39 ± 7.66
Condensed tannin (mg/g)	55.58 ± 9.77	97.78 ± 5.74 *	127.54 ± 6.97 *	107.33 ± 6.38 *

All data are expressed as the mean ± standard deviation of at least three experiments. The statistical results are shown as significant differences (* *p* < 0.05) from HPT-BW. Abbreviations: HPT, Hakka pomelo tea; BW, boiling water; HW, hot water reflux; Alc, ethanol; EA, ethyl acetate.

**Table 2:** The gallic acid and hesperidin content in HPT alcohol or ethyl acetate extracts.

	HPT-EA	HPT-Alc
Gallic acid (mg/g)	204.83 ± 18.93 *	103.94 ± 9.32
Hesperidin (mg/g)	41.35 ± 5.23 *	25.79 ± 2.38

All data are expressed as the mean ± standard deviation of at least three experiments. The statistical results are shown as significant differences (* *p* < 0.05) between HPT-Alc and HPT-EA. Abbreviations: HPT, Hakka pomelo tea; Alc, ethanol; EA, ethyl acetate.

**Table 3:** The combination index (R value) of gallic acid and hesperidin for HT-29 cells.

Hesperidin (μg/mL)	Gallic Acid (μg/mL)			
	10	20	50	100
10	0.85	0.92	0.93	1.07
20	1.02	1.19	1.37	1.18
50	1.12	1.29	1.43	1.09
100	0.91	1.00	1.18	0.87
200	0.68	0.81	0.87	0.71

An expected value of cell survival, S-exp, was defined as the product of the survival observed for gallic acid alone and the survival observed for hesperidin alone: S-exp = (S-gallic acid) × (S-hesperidin). The actual survival observed for the combination of gallic acid and hesperidin was defined as S-obs. The combination effect was calculated as R value according to the equation of synergistic ratio: R = (S-exp)/(S-obs). Synergy was defined as any value of R greater than unity. Values of R of 1.0 or less indicated an absence of synergy.

## Data Availability

All data used to support the findings of this study are available from the corresponding author, Hsu, C.-P., upon reasonable request. Corresponding author’s email: hsucp@mail.ypu.edu.tw.

## Supplementary Materials

The following supporting information can be downloaded at: https://www.mdpi.com/article/10.3390/cimb45060312/s1. Figure S1 contains the representative chromatography results of HPLC analysis by injecting different dose of gallic acid and hesperidin, and the standard curve calculated from the quantified results of each chromatograph. Figure S2 contains the representative chromatography results of HPLC analysis by injecting HPT-Alc (A) and –EA (B).
