# Supplementary figures and images for: Synergistic Chemopreventive Effects of a Novel Combined Plant Extract Comprising Gallic Acid and Hesperidin on Colorectal Cancer
Source: Curr Issues Mol Biol. 2023 Jun 5;45(6):4908–22. doi: 10.3390/cimb45060312 (PMC10297232; doi:10.3390/cimb45060312)

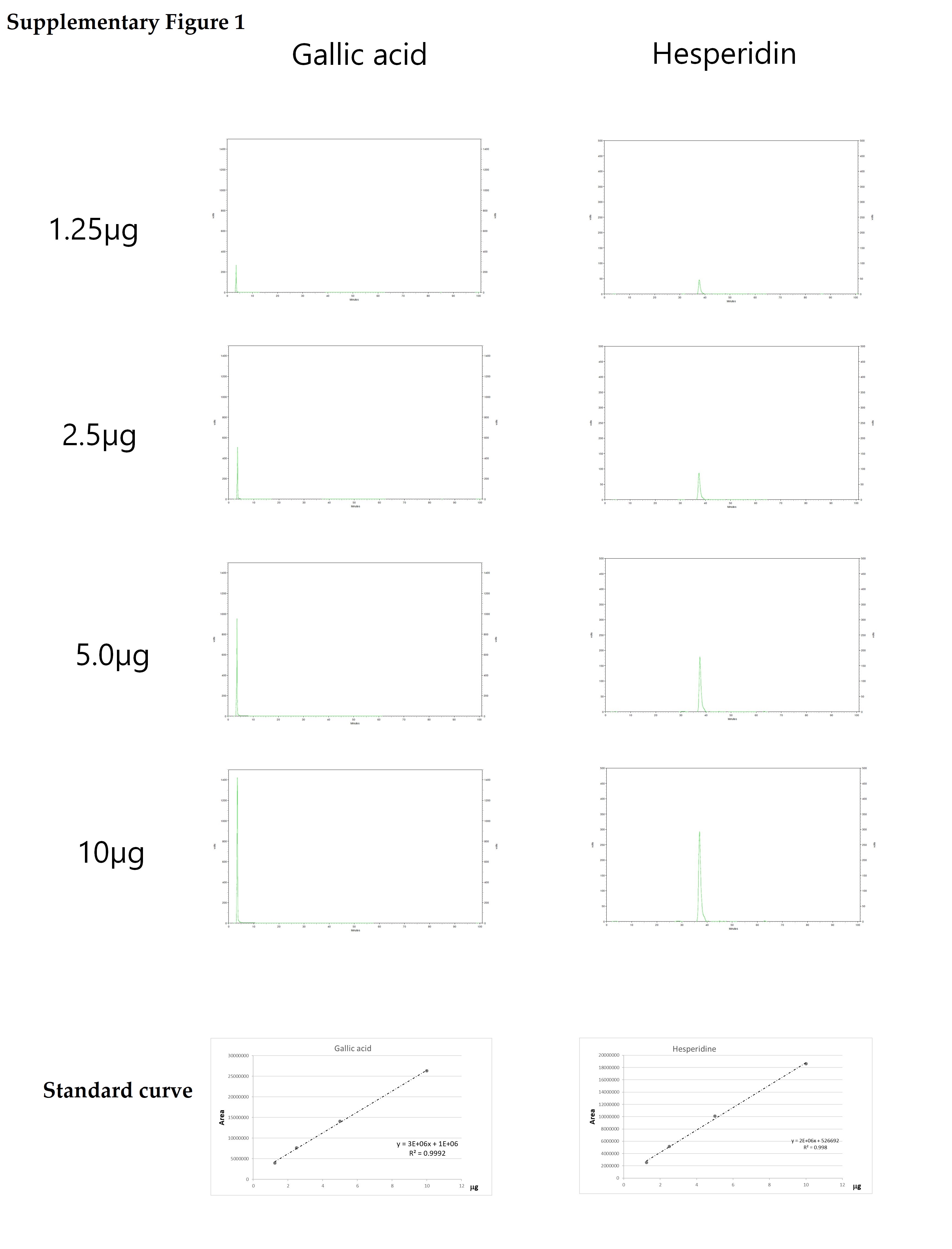

Supplement: Supplementary file 1 [file cimb-45-00312-s001.zip › Figure S1.jpg]

A.

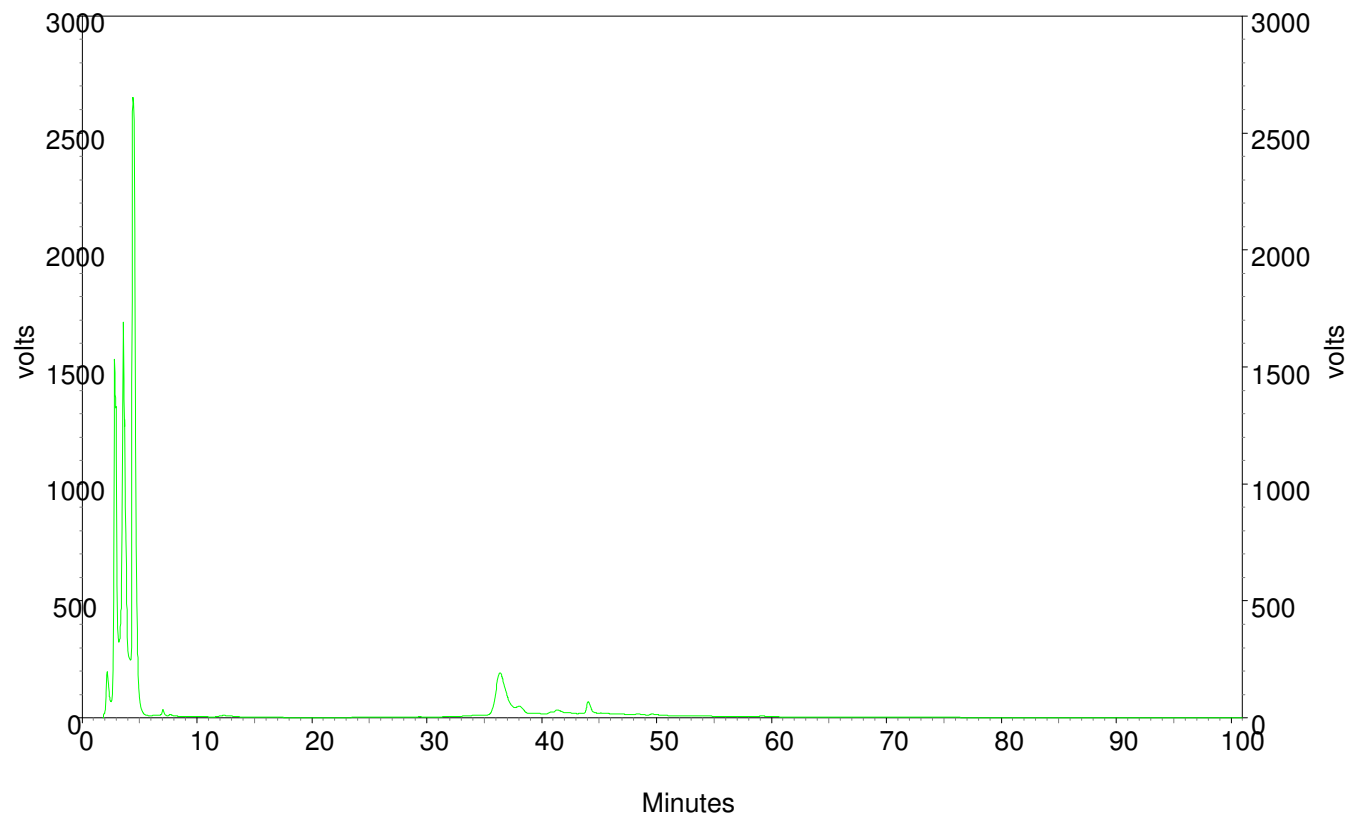

B.

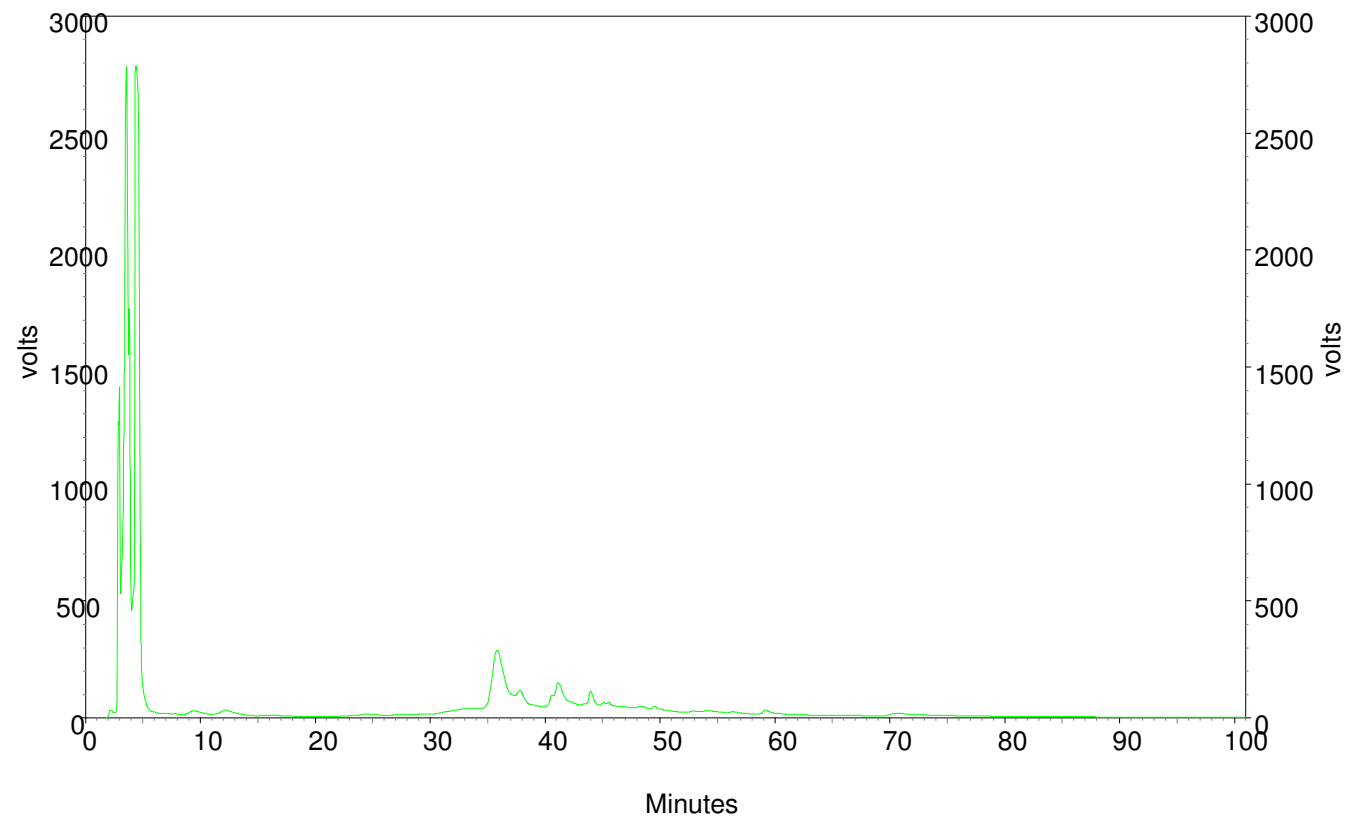

Supplement: Supplementary file 1 [file cimb-45-00312-s001.zip › Figure S2.pdf]
